# Dietary patterns and micronutrients in respiratory infections including COVID-19: a narrative review

**DOI:** 10.1186/s12889-024-18760-y

**Published:** 2024-06-21

**Authors:** Zahra Salehi, Mohammadreza Askari, Alireza Jafari, Batoul Ghosn, Pamela J. Surkan, Mohammad Javad Hosseinzadeh-Attar, Hamed Pouraram, Leila Azadbakht

**Affiliations:** 1 Department of Community Nutrition, School of Nutritional Sciences and Dietetics, Tehran University of Medical Sciences, Tehran, IR Iran; 2 Department of International Health, Johns Hopkins Bloomberg School of Public Health, Baltimore, MD USA; 3 Department of Clinical Nutrition, School of Nutritional Sciences and Dietetic, Tehran University of Medical Sciences, Tehran, Iran; 4 Department of Nutrition and Biochemistry, School of Public Health, Tehran University of Medical Sciences, Tehran, IR Iran; 5 Diabetes Research Center, Endocrinology and Metabolism Clinical Sciences Institute, Tehran University of Medical Sciences, Tehran, IR Iran; 6 Department of Community Nutrition, School of Nutrition and Food Science, Isfahan University of Medical Sciences, Isfahan, IR Iran

**Keywords:** Food intake, Serum levels, Respiratory infections, Influenza, Pneumonia, Acute respiratory syndrome, COVID-19

## Abstract

**Background:**

COVID-19 is a pandemic caused by nCoV-2019, a new beta-coronavirus from Wuhan, China, that mainly affects the respiratory system and can be modulated by nutrition.

**Methods:**

This review aims to summarize the current literature on the association between dietary intake and serum levels of micronutrients, malnutrition, and dietary patterns and respiratory infections, including flu, pneumonia, and acute respiratory syndrome, with a focus on COVID-19. We searched for relevant articles in various databases and selected those that met our inclusion criteria.

**Results:**

Some studies suggest that dietary patterns, malnutrition, and certain nutrients such as vitamins D, E, A, iron, zinc, selenium, magnesium, omega-3 fatty acids, and fiber may have a significant role in preventing respiratory diseases, alleviating symptoms, and lowering mortality rates. However, the evidence is not consistent and conclusive, and more research is needed to clarify the mechanisms and the optimal doses of these dietary components. The impact of omega-3 and fiber on respiratory diseases has been mainly studied in children and adults, respectively, and few studies have examined the effect of dietary components on COVID-19 prevention, with a greater focus on vitamin D.

**Conclusion:**

This review highlights the potential of nutrition as a modifiable factor in the prevention and management of respiratory infections and suggests some directions for future research. However, it also acknowledges the limitations of the existing literature, such as the heterogeneity of the study designs, populations, interventions, and outcomes, and the difficulty of isolating the effects of single nutrients from the complex interactions of the whole diet.

## Introduction

The significance of nutrition cannot be overstated when it comes to its impact on respiratory diseases. It is well-documented that nutrition has a profound influence on the immune system, which in turn affects the respiratory system [1]. This relationship has been confirmed by numerous studies conducted over the years [2–4]. Given the critical role of nutrition, it is imperative to further investigate its effects on respiratory health. This study aims to contribute to this body of knowledge and underscore the necessity of continued research in this area.

The role of nutrition in modulating the immune and respiratory systems and influencing the susceptibility and severity of COVID-19 has been a topic of interest for many researchers. Nutrition may affect the host response to viral infections, as well as the viral replication and transmission [5]. Dietary intake and serum levels of micronutrients, malnutrition, and dietary patterns may have an impact on the prevention, progression, and recovery of COVID-19, as well as on the long-term complications and sequelae of the infection [6–10]. However, the evidence on the relationship between nutrition and COVID-19 is still emerging and inconclusive, and there are many gaps and challenges in the existing research.

The research on the relationship between food intake and serum levels of nutrients with coronavirus is limited. The observations regarding dietary intake and nutrient serum levels in relation to other respiratory infections, such as SARS, Middle East Respiratory Syndrome (MERS), influenza, seasonal colds and lung infections may be like those between dietary intakes and nutrient serum levels and COVID-19 [11]. Six review articles have been conducted in this area. One of these articles focused on clinical trials related to viral infections [12]. Another review summarized management strategies for critically ill patients [13]. Additionally, one review gathered information on effective pharmaceutical and nutritional treatments [14]. Furthermore, a study investigated the therapeutic effects of nutrients in boosting the immune system [15]. Moreover, another review provided guidance on hygiene and nutritional principles [16]. Finally, a systematic review encompassed all studies evaluating the role of dietary patterns and nutrients in immune system function and viral infections (Corona and MERS) [17].

Micronutrients play a crucial role in supporting the immune system, especially in the context of COVID-19 [18]. A balanced diet rich in essential nutrients like vitamin D, vitamin A, B vitamins (folate, vitamin B6 and vitamin B12), vitamin C, and minerals such as iron, copper, selenium, and zinc contribute to the normal functions of the immune system [18]. Deficiencies or even suboptimal intakes of these micronutrients in targeted groups of patients and in distinct and highly sensitive populations could potentially weaken the immune system, thereby increasing susceptibility to COVID-19 [18]. For instance, zinc and vitamins C and D are micronutrients with robust evidence of their immunomodulating activity, such that their deficiency, even if marginal, can compromise metabolism and, consequently, their action on the immune system [19]. It is important to note that while a balanced diet can help strengthen the immune system, it will not prevent or cure COVID-19 infection. Frequent handwashing and social distancing remain critical to reduce transmission. The relationship between micronutrients and COVID-19 is still being explored, and further research is needed to fully understand this complex interaction.

The COVID-19 pandemic has significantly impacted dietary patterns across various population groups [20]. The lockdown measures implemented in many countries have restricted access to fresh food and limited physical activity, leading to changes in eating habits [20]. For instance, a study conducted in Saudi Arabia found that the quarantine measures affected dietary patterns, with changes observed in the type and frequency of snack consumption, the main meal-type, and a significant increase in fluids consumption [21]. Moreover, a dietary pattern characterized by healthy plant-based foods was associated with a lower risk and severity of COVID-19 [22]. The relationship between dietary patterns and COVID-19 is complex and multifaceted, warranting further exploration. It is crucial to maintain a balanced diet rich in essential nutrients to support the immune system and potentially mitigate the impact of COVID-19.

The scope of this study is to explore the relationship between dietary intake, micronutrient serum levels, malnutrition, dietary patterns, and respiratory infections, with a particular focus on COVID-19. The need for this study arises from the ongoing COVID-19 pandemic and the increasing evidence suggesting a link between nutrition and immune response, particularly in relation to respiratory-related symptoms, which are the most common cause of COVID-19 mortality. This study aims to fill a gap in the current literature by examining a wide range of dietary factors, including specific micronutrients (vitamins A, E, D, and C, zinc, magnesium, iron, omega-3 fatty acids, probiotics), malnutrition, and overall dietary patterns. It also seeks to address the limited research on the impact of certain dietary components, such as omega-3 and fiber, on COVID-19 prevention. The findings of this study could potentially inform dietary recommendations for the prevention of COVID-19 and other respiratory infections, contribute to the development of public health strategies during the pandemic, and guide future research in this area. This study is therefore both timely and necessary in the face of the ongoing global health crisis.

Therefore, we conducted a narrative review to summarize and present the available literature on the association between dietary intake and serum levels of micronutrients, malnutrition, and dietary patterns and respiratory infections, including flu, pneumonia, and acute respiratory syndrome, with a focus on COVID-19. We aimed to provide a comprehensive overview of the topic, and to track the development of the scientific and clinical concepts related to nutrition and respiratory infections. We also aimed to identify the strengths and weaknesses of literature, and to suggest some directions for future research.

## Methods

### Study framework

We aimed to review the current literature on the association between dietary intake and serum levels of micronutrients, respiratory infections, influenza, pneumonia, acute respiratory syndrome, and corona viruses, with a focus on COVID-19. We searched for relevant articles in various scientific databases and selected those that met our inclusion criteria.

### Search strategy

We used MeSH terms (medical subject headings) and other related keywords: “Novel coronavirus 2019” or “2019 nCoV” or “COVID-19” or “Wuhan coronavirus” or “Wuhan pneumonia” or “SARS-CoV-2” or “severe acute respiratory syndrome coronavirus 2” or “respiratory disease” or “respiratory infection” or “acute lower respiratory tract infections” or “lung infection” or “influenza” or “COPD” or “inflammatory response” or “pneumonia” or “common cold” or “sepsis” or “acute respiratory distress syndrome” or “severe acute respiratory syndrome-related coronavirus” or “bronchitis” or “chronic obstructive pulmonary disease” or “obstructive pulmonary disease” AND “vitamin D” or “vitamin A” or “carotenoids” or “zinc” or “vitamin” or “selenium” or “folic acid” or “vitamin B” or “vitamin E” or “vitamin B12” or “cobalamin” or “thiamine” or “riboflavin” or “niacin” or “pantothenic acid” or “pyridoxine” or “biotin” or “folic acid” or “cobalamin” or “amino acid” or “omega 3” or “water” or “malnutrition”.

### Inclusion and exclusion criteria

The inclusion criteria for the study were: 1) examination of the relationship between dietary intake and serum levels of nutrients with respiratory infections, influenza, pneumonia, or acute respiratory syndrome with a focus on coronavirus, and 2) all observational studies. The review excluded other types of human and animal studies, in vitro studies, irrelevant sources, and studies not published in English.

### Data extraction

Database searches as well as reference extraction were performed by two separate investigators. In prospective studies, dietary intake and serum levels of nutrients measured at the beginning of the study were considered exposure variables, while respiratory infections, influenza, pneumonia, and acute respiratory syndrome were defined as outcome variables. Information was extracted on the first author’s name, year of publication, location of the study, age range of participants (in cohort studies at the start or in cross-sectional studies), gender, sample size, follow up duration (in prospective studies), number of participants who developed respiratory infections, influenza, pneumonia and acute respiratory syndrome during the study, person-years, and any adjustments made for confounding variables.

## Results

A total of 292 studies were included in the review.

### Dietary patterns

Healthy dietary patterns have been shown to activate the immune system through the gut microbiota [23–26]. A meta-analysis showed that a healthy dietary pattern was associated with lower prevalence of chronic obstructive pulmonary disease (COPD) [27]. To the best of our knowledge, there are no original research articles that have examined the causal effect of dietary pattern on COVID-19 prevention, symptoms, or mortality using rigorous methods such as randomized controlled trials.

### Vitamin D

Several studies have indicated a relationship between vitamin D deficiency and an increased susceptibility to respiratory viral infections [28–30]. A meta-analysis of eight observational studies in adults found an association between vitamin D deficiency (VDD) and an increased risk of community-acquired pneumonia (CAP) [31]. Subsequently, seven original articles produced similar results [32–38]. A meta-analysis of six observational studies showed vitamin D deficiency was prevalent in patients with recurrent tonsillitis [39].

A meta-analysis of ten cohort studies in pregnant women showed no strong association between early life vitamin D status and the risk of developing respiratory tract infections (RTIs) in infants [40]. Of the 15 original studies published since then, four produced similar results while the rest produced conflicting conclusions [41–55]. There is currently a meta-analysis on vitamin D in relation to acute viral airway infections in healthy adults which analyzed individual participant data from randomized controlled trials (RCTs). It found that vitamin D supplementation reduced the risk of acute respiratory tract infections among all participants [56]. However, a prospective cohort study found that serum concentration of 25- hydroxyvitamin D was associated with the incidence of acute viral respiratory infections [57]. Vitamin D deficiency in elite swimmers was also associated with increased acute upper respiratory tract infections [58]. Serum levels of vitamin D were also found to be a predictor of bronchitis [59] and an association was seen between symptoms of upper respiratory tract illness (URTI) and vitamin D deficiency in adults [60] and between low 25OHD levels and acute respiratory infections (ARI) in children [29]. No association was found between vitamin D and COPD [61]. There is currently no available meta-analysis on this topic.

A prospective study found that children with active tuberculosis had significantly lower vitamin D levels [62] but there has been no meta-analysis on this topic to date.

A meta-analysis of critically ill children with sepsis found that vitamin D deficiency was linked to increased mortality [63]. Seven subsequent articles confirmed these findings [54, 64–69]. Two original articles have found a significant association between low 25 (OH) D levels and mortality in critically ill patients [70] and a link between high vitamin D levels and reduced organ dysfunction [71]. No meta-analysis has been performed on vitamin D and subclinical interstitial lung disease (ILD) in adults, but Kim et al. found an association between vitamin D deficiency and subclinical ILD [72]. No meta-analysis has been performed for the relationship between cystic fibrosis in children and vitamin D, but Oliveira et al. (2019) found no connection between the severity of lung disease in cystic fibrosis group and vitamin D levels [73]. There has been meta-analysis on the relationship between respiratory disease and vitamin D in adults but low serum levels of 25 (OH) D were found to be associated with respiratory disease in the elderly [74].

Vitamin D deficiency has been identified as a risk factor for COVID-19 [75–79] With mean level of vitamin D being inversely associated with SARS-CoV-2 infection and fatality in the Indian population [80]. Studies have linked vitamin D deficiency to mortality from COVID-19 [81–83]. Vitamin D deficiency also impacted the severity and hospitalization of COVID-19 in China [84] and was associated with COVID-19 patient outcomes [85]. However, a study found no association between 25 (OH) D concentration and chronic inflammation, impaired pulmonary function tests, pathological outcomes on CT scans, or persistent symptoms [86]. Vitamin D deficiency was more prevalent in critically ill ICU patients infected with coronavirus [87] and several studies reported lower levels of vitamin D in hospitalized COVID-19 patients [88–99]. However, one study found no potential association between vitamin D concentrations and COVID-19 infection risk [100].

### Vitamin E

Vitamin E supports the immune system through antioxidant activity [101, 102]. Regarding recurrent respiratory infection (RRI) in children, no meta-analysis on Vitamin E and RRI has been conducted. However, one study found a positive association between vitamin E deficiency and RRI [45]. No meta-analysis on the relationship between antioxidants and COPD has been published. In terms of serum levels, several studies have found that people with COPD had lower antioxidant status [103–105]. The benefits of higher serum concentration s of antioxidants on lung health have been shown in men [106, 107]. An imbalance between oxidants and antioxidants has been found in patients with COPD [108] but not in all studies [109]. There was no significant relationship between plasma Vitamin E levels and COPD severity [110]. A positive association between tocopherols and pulmonary gas diffusion was observed only in patients with lung disease [111]. Adherence to high antioxidant dietary patterns such as the Dietary Approaches to Stop Hypertension (DASH) diet, was found to be lower in lower in patients with COPD [112]. Vitamin E and olive oil intake were linked to reduced oxidative stress in current smokers with COPD [113]. A positive association was observed between a high intake of three antioxidants (vitamin C, vitamin E, and â-carotene) and pulmonary function, but this disappeared after adjusting for energy intake [114]. Other studies have shown an inverse association between diet and serum antioxidant levels and COPD [115–118].

### Vitamin A

Vitamin A has been shown to have anti-infection properties in several studies [119–122]. A meta-analysis of 62 observational studies in children, supports the beneficial effects of vitamin A on infection [123]. However, subsequent research has produced conflicting results [124–135].

Studies have shown a positive association between vitamin A deficiency and respiratory infection in children [136–154]. Three studies showed an association between infection severity and vitamin A deficiency [45, 149, 155, 156]. Importance of vitamin A has been indicated in several studies [130, 157, 158] but some research has produced conflicting results [159–161].

No meta-analysis has been published on the relationship between vitamin A and pneumonia in children. Two studies have shown an association between lower serum vitamin A level and increased risk of pneumonia [162, 163]. Some studies have found that children with pneumonia experience a temporary decrease in vitamin A levels [146–149, 152]. The relationship between serum retinol levels and increased risk of pneumonia has produced conflicting results in different studies [160, 164].

### Iron

Iron is necessary for the function of the immune system [165]. Iron deficiency can impair host immunity and excess iron can cause oxidative stress, which increases the risk of harmful viral mutations [166, 167]. A meta-analysis of 41 cohort studies found that anemia is prevalent among tuberculosis (TB) patients [168].

There is no meta-analysis on the relationship between viral infection and iron in children. However, there have been four individual studies on this topic. Two studies found that during viral infection, serum hepcidin levels increased and iron levels decreased [169, 170]. Blood plasma transferrin saturation by iron was significantly reduced in patients with severe forms of influenza stomatitis [171]. The mean hemoglobin level in infants and toddlers decreased with increasing numbers of infectious episodes [172].

Several studies have shown that ferritin is an indicator of severity and outcome of the disease. Serum ferritin levels are elevated in severe cases of COVID-19 [173–177]. Two cross-sectional studies found similar results, including one study on diabetic people with coronavirus [178]. Ferritin is closely related to disease severity, along with D-dimer [179].

### Zinc

Zinc plays a significant role in the protecting immune function [180]. A meta-analysis of 32 observational studies in pregnant women found a protective effect of zinc against childhood wheezing [181]. Two later studies also found similar protective effects [182, 183].

Although no meta-analysis exists for the connection between zinc and acute lower respiratory tract infections in children, two original articles suggest that zinc plays a role in these infections [184, 185]. Though no meta-analysis has been performed, one study discovered that zinc levels in children can aid in diagnosing and predicting the outcome of pneumonia [186].

### Selenium

Selenium has both antioxidant and anti-inflammatory properties [14, 187]. The level of maternal selenium exposure experienced by the fetus may impact the risk of wheezing [188]. The concentration of selenium in cord blood has been linked to the occurrence of allergic rhinitis in children [183]. Prenatal exposure to selenium has been linked to wheezing in childhood [189]. To the best of our knowledge, no study has investigated the relationship between selenium and coronavirus.

### Magnesium

Magnesium has a significant impact on immune function [190]. No meta-analysis has been conducted in the fiend of nutrition to examine the relationship between magnesium and respiratory diseases using observational studies. In adults with COPD, serum magnesium levels have been found to be directly associated with quality of life (QOL) [61].

Maternal dietary magnesium intake during pregnancy may reduce the risk of eczema in children [191]. Childhood dietary habit have a crucial role in the development of wheezing disease [192]. Two studies have found that low magnesium intakes is associated with an increased risk of hyperreactivity during seasonal allergies [193, 194].

### Beta-carotene

Beta-carotene is a precursor for vitamin A synthesis, which is derived from plants [14]. No further studies on this topic have been published to date.

### Malnutrition

Nutrition is a critical factor affecting the immune response [195]. A meta-analysis based on 54 observational studies on children under 5 years old showed that malnutrition is linked to increased deaths from ALRI [196]. A 2020 meta-analysis of 12 observational studies found a direct association between malnutrition and pneumonia in children [197].

We could not find any meta-analysis examining the relationship between malnutrition and adult pneumonia. However, nutritional care after general and digestive surgery may prevent postoperative pneumonia in malnourished patients [198] as shown by several studies that associate malnutrition with incidence of pneumonia, including in hospitalized patients [199–210]. Some studies have indicated that the mortality rate increases with higher degree of malnutrition among patients with pneumonia [211–214]. Two studies demonstrated the importance of nutritional status in the prevention and treatment of pneumonia [214, 215]. However, malnutrition did not play a significant role in the incidence of pneumonia in hospitalized elderly patients [216]. A study by Kelaiditi et al. in 2014 found that 58.7% of elderly participants were at risk of malnutrition [217].

This information suggests that there is limited research available on the relationship between malnutrition and RNA viruses in children, with only five studies found. One of these studies found a connection between underweight children and lower serum antibody titers [218]. In another study, malnutrition was initially associated with acute respiratory infection; but after adjusting for covariates that could have affected the results, this association was no longer present [219]. In another study, the prevalence of viral infections increased as the severity of malnutrition increased [220]. A study of premature infants showed that malnutrition was not a significant contributor to respiratory failure (RF) [221]. However, malnutrition was found to be risk factor for mortality in children hospitalized due to respiratory influenza A H1N1 virus infections [222]. There has not been meta-analysis on the relationship between sepsis and malnutrition in adults, however, there are conflicting results [223].

There has not been no meta-analysis on the relationship between antiviral immunity and malnutrition in children. In a study, higher mortality was observed in children with TB-HIV co-infection and severe malnutrition [224]. Additionally, there has not been a meta-analysis on the relationship between tuberculosis and adult malnutrition, but 44 studies have been conducted on this topic. Several studies have shown that malnutrition is highly prevalent among patients with active pulmonary tuberculosis [225–234]. Undernutrition was found to be one of the most common comorbidities among young tuberculosis patients [235]. Two studies indicated that a majority of patients with pulmonary tuberculosis (PTB) were suffering from nutritional deficiencies at the onset of treatment ([236, 237]. Serum albumin levels were negatively associated with C-Reactive-Protein (CRP) levels [238]. In some studies malnutrition has been found to be associated with an increased risk of developing pulmonary tuberculosis (PTB) [239–246]. Several studies have indicated that low body mass index (BMI) and malnutrition to be associated with tuberculosis [247–264]. There has been no meta-analysis on the association between tuberculosis and malnutrition in children. However, one study did not find an increased risk of mortality from tuberculosis in severely malnourished children [265]. Several studies have shown an association between malnutrition and TB [266–272].

Patients with both COVID-19 and malnutrition had a higher inflammatory response, greater acute heart damage and weaker immune functions. Malnutrition was significantly related to poor outcomes in COVID-19 outcomes, while patients with normal nutritional status had better prognosis in terms of white blood cell count, inflammatory status, and mortality [273].

### Omega 3 fatty acids

Long-chain polyunsaturated fatty acids (PUFAs) play important roles as both pro-inflammatory and anti-inflammatory factors [274]. A meta-analysis of 23 observational studies in children found that consuming fish had a beneficial effect in reducing wheezing [275]. No meta-analysis has been published for adults, but there has been one case–control study which showed that regular dietary intake of fish oil did not effectively suppress a special bronchial response [276].

### Cobalamin

Cobalamin plays an essential role in supporting the immune system by aiding in the production of white blood cells [277, 278]. A meta-analysis of nine observational studies in adults did not support the hypothesis that vitamin B12 and folate levels are causally linked to hay fever or allergy biomarkers [279].

### Fiber

Dietary fibers can enhance immune function primarily by producing Short Chain Fatty Acids (SCFA) [279–281]. Currently, no meta-analysis exists on the relationship between COPD and fiber. In a cross-sectional study, Butler et al. found that a diet high in fiber from fruits (and possibly soy foods) may decrease the incidence of acute respiratory symptoms [282]. Hirayama et al. observed an inverse relationship between vegetable intake and the risk of COPD in Japanese adults [283]. Two studies found that high fiber consumption was inversely associated with the incidence of COPD in men who were current or former smokers [284, 285]. A study indicated that dietary fiber was independently linked to better lung function and reduced prevalence of COPD [286]. Another study suggested that a diet high in fiber, especially cereal fiber, may lower the risk of developing COPD [287]. In a case control study, the medium intake of dietary fiber in the COPD group was notably lower than the average intake (6.14 vs. 8.45 g / day, *p* < 0.001) [288].

## Discussion

This review aimed to understand the association between dietary intake, serum levels of micronutrients, malnutrition, dietary patterns, and respiratory infections, with a specific emphasis on COVID-19. The results indicate that dietary patterns, malnutrition, and certain nutrients such as vitamins D, E, A, iron, zinc, selenium, magnesium, omega-3 fatty acids, and fiber may play a significant role in preventing respiratory diseases, alleviating symptoms, and lowering mortality rates. However, the evidence is inconsistent and inconclusive, necessitating further research to clarify the mechanisms and optimal doses of these dietary components. The impact of omega-3 and fiber on respiratory diseases has been primarily studied in children and adults, respectively, and few studies have examined the effect of dietary components on COVID-19 prevention, with a greater focus on vitamin D. These inconsistencies may be due to the heterogeneity of the study designs, populations, interventions, and outcomes, and the difficulty of isolating the effects of single nutrients from the complex interactions of the whole diet. We are not aware of any other reviews that have addressed this specific question, although there are some reviews that have examined the effects of nutrition on other respiratory infections or on COVID-19 outcomes.

Fish and seafood are excellent sources of fatty acids [289] that have anti-inflammatory effects through the G protein-coupled receptor 120 (GPR 120) [290] and resolvin E1 (RvE1) [291]. These foods are rich in zinc, copper, and selenium, which play a role in antioxidant enzymatic mechanisms [292–295]. Whole grains have anti-inflammatory and antioxidant properties [292, 293]. In addition, fruits, vegetables, and whole grains rich in fiber can have antioxidant effects through the production of SCFAs, including butyrate, by gut microbiota through fiber fermentation [294].

One of the mechanisms connecting the high prevalence of low levels of vitamin D in critically ill patients with sepsis is frequent reduction in serum concentrations of vitamin D-transporting proteins [296]. Vitamins are involved in regulating the production of antimicrobial peptides (AMPs) such as β-defensin and catalystidine [297, 298]. Vitamin D increases AMP production, which is effective against a wide range of fungi and bacteria [299, 300].

The results regarding vitamin D and respiratory tract infections in infants have been contradictory. Vitamin D seems to be crucial for the responses of interferon-g dependent T cells to infection and important for activating TLR and antimicrobial responses [301].

Regarding the association between vitamin D and COPD in adults, contrary to cross-sectional studies, a cohort study found this association [302]. Vitamin D status has been shown to be inversely related to inflammatory biomarkers [303–305] which may have a pathogenesis role in COPD [306]. Vitamin D directly regulates epithelium function as several types of epithelium express vitamin D receptors and to respond to vitamin D. Vitamin D may also indirectly modulate the epithelial cell function in the lung by acting on inflammatory cells [307, 308].

In a study that investigated the link between vitamin E and recurrent respiratory infections in children, it was found that vitamin E levels was significantly lower in both the active and stable recurrent respiratory infection groups and significantly lower in the active cohort compared to the stable group [45]. Vitamin E stabilizes the cell membrane structure of the and its supplementation enhances cellular immunity [56].

Regarding pneumonia, seven studies found negative associations between vitamin A and pneumonia in children [146–149, 151, 162, 163]. One study found a positive association [164], while another found no association [160]. A study showed that increased concentrations of serum retinol were significantly correlated with increased pneumonia risk [164]. Several studies conducted in lower- and middle-income countries have identified poor zinc and vitamin A status as risk factors for pneumonia and lower respiratory tract infections [137, 309].Vitamin A has a significant pleiotropic role in protecting the normal mucosal barrier [310].

In existing studies of viral infections and iron in children, decreased levels of serum hemoglobin, iron and plasma transferrin saturation were observed during infection [169–172]. The decrease in serum iron levels during infection may be due to increased hepcidin [311–316].

Additionally, zinc deficiency affects the survival, reproduction and maturation of immune cells that plays a role in both innate and adaptive immunity [317].

One study found an association between serum selenium levels and childhood wheezing but found no association between dietary selenium intake and wheezing. The estimates of dietary intake of selenium are not entirely reliable when using the food frequency questionnaires (FFQ), because food nutrient tables that use both FFQs and weighted records do not account for the wide diversity in the selenium content of foods due to geographical differences in soil selenium [189].

Since magnesium causes relaxation in smooth muscle and restricts its ability to contract, it may impact COPD-related quality of life by improving respiratory symptoms [318, 319]. Findings from studies on pneumonia and malnutrition in children showed a direct association [197]. It appears that malnutrition weakens the respiratory muscles making it unable to clear the airways of secretions and weakens the immune system [320].

Studies in adults have also supported the effect of malnutrition on pneumonia [198, 199, 210, 211, 214, 215, 217, 321, 322] with only one study showing results to the contrary [217]. In addition, hypoalbuminemia and low BMI are correlated with mortality in senile pneumonic patients [323]. Albumin influences the host’s defense mechanisms against bacterial infection through the complement function and defensin production [324, 325]. Therefore, nutritional therapy may partially reduce risk factors associated with malnutrition and help improve defense mechanisms against bacterial infection, reducing the development of pneumonia [203].

In Hong et al.’s study, inadequate nutrition and an impaired immune system did not have a synergistic impact on mortality in acute septic patients. Caloric restriction, by regulating inflammatory pathways, has been shown to increase cell survival in mammals [326]. Caloric restriction during the acute phase of infection may reduce the inflammatory response and damage by regulating hormonal, inflammatory, and metabolic pathways. Caloric restriction has also been linked to better glycemic control [326, 327], because short-term hyperglycemia can disrupt the body’s natural immune responses to infection [328].

Regarding the consumption of fiber and COPD, a diet rich in fiber has been found to be inversely associated with the incidence and symptoms of COPD [282–288]. This might be due to fiber’s antioxidant and anti-inflammatory properties [329–337]. Fiber consumption has been linked to decreased C-reactive protein level, a marker of systemic inflammation. Fiber may modulate inflammation through several mechanisms, including by slowing glucose absorption [338] and reducing lipid oxidation [329], or affecting the production of anti-inflammatory cytokines in the gut flora [339]. Some components of fiber, such as trace elements or related nutrients like flavonoids, may have a positive impact on the lungs [340], however, the fiber sources in different studies have varied, which may be due to limited diversity of fiber sources in some populations, for example, white rice is the main grain in Asian diets whereas whole grains are found in some Western diets [341].

Observational studies on SARS-CoV-2 are limited, with most of them focusing on vitamin D [342]. Dysregulation of the renin-angiotensin system is also one of the early mechanisms of lung damage in COVID-19 [343, 344]. Vitamin D upregulates anti-inflammatory intermediates, which is critical when considering the excessive inflammatory response triggered by COVID-19 in the immune system [345]. However, A cohort study [346], found that low levels of vitamin D in most COVID-19 patients may be due to insulin resistance diabetes, overweight, or obesity, as these are of risk factors for both low vitamin D levels and for COVID-19 [347, 348].

Poor nutritional status may contribute to the increased mortality in COVID-19 patients [349]. Most COVID-19 patients have elevated CRP levels [349, 350]. Inflammation and malnutrition often occur simultaneously because malnutrition can increase susceptibility to infection, while infections can lead to malnutrition by increasing nutrient needs and reducing appetite [351].

### Strengths and limitations

We found that the literature on the association between dietary intake and serum levels of micronutrients, respiratory infections, influenza, pneumonia, acute respiratory syndrome and COVID-19 was varied and rich, but also faced some methodological and conceptual limitations. Most of the articles we reviewed had an acceptable to good quality, but they differed in their study designs, populations, interventions, and outcomes, which made it difficult to compare and synthesize their results. Therefore, we were unable to conduct a meta-analysis on this topic, and we had to rely on a narrative synthesis to present the main findings and trends. Another limitation of our study was that we focused only on viral infections related to the respiratory tract, as COVID-19 primarily affects the respiratory system. This may have excluded some relevant studies that examined the effects of nutrition on other types of viral infections, such as gastrointestinal or systemic infections. Future studies should consider these limitations and aim to provide more comprehensive and robust evidence on the relationship between nutrition and COVID-19, by using more standardized and rigorous methods, exploring the mechanisms and the dose–response relationships of dietary components, and including a wider range of viral infections and outcomes.

## Conclusion

In this systematic review, we examined the relationship between dietary intake and serum levels of micronutrients, malnutrition, and dietary patterns and respiratory infections, with a focus on COVID-19. We found that dietary patterns, malnutrition, and certain nutrients such as vitamins D, E, A, iron, zinc, selenium, magnesium, omega-3 fatty acids, and fiber play a significant role in preventing respiratory diseases, alleviating symptoms, and lowering mortality rates. However, we also identified some limitations and gaps in the existing literature, such as the lack of randomized controlled trials, the heterogeneity of study designs and populations, the confounding effects of other factors, and the scarcity of studies on the specific effect of dietary components on COVID-19 outcomes. Therefore, we suggest that future research should conduct more rigorous and comprehensive studies to test the causal effect of dietary patterns on COVID-19 prevention, symptoms, or mortality, and to explore the underlying mechanisms and pathways of how nutrition influences the immune and respiratory systems. Adopting a healthy and balanced diet, rich in certain micronutrients and fiber, may be a feasible and effective strategy to protect against respiratory infections, including COVID-19, and to improve the overall health and well-being of individuals and populations.

## Data Availability

Not applicable.

## Abbreviations

TUMS: Tehran University of Medical Science
COVID-19: Coronavirus disease 2019
SARSr-CoV-2: Severe Acute Respiratory Syndrome Related Coronavirus
MERS: Middle East Respiratory Syndrome
ICU: Intensive Care Unit
RCT: Randomized Controlled Trials
MeSH: Medical Subject Headings
COPD: Chronic Obstructive Pulmonary Disease
RSV: Respiratory Syncytial Virus
VDD: Vitamin D Deficiency
CAP: Community Acquired Peumonia
RTI: Respiratory Tract Infection
URTI: Upper Respiratory Tract Illness
ILD: Interstitial Lung Disease
RRI: Recurrent Respiratory Infection
DASH: Dietary Approaches to Stop Hypertension
ALRI: Acute Lower Respiratory Tract Infection
TB: Tuberculosis
QOL: Quality of Life
FEF: Forced Expiratory Flow
PTB: Pulmonary Tuberculosis
PUFA: Polyunsaturated Fatty Acid
SCFA: Short Chain Fatty Acid
IL: Interleukin
GPR 120: G Protein-coupled Receptor 120
RvE1: Resolvin E1
VDR: Vitamin D Receptor
TLR: Toll-like receptor
AMP: Antimicrobial Peptide
Mtb: Mycobacterium tuberculosis
FEV1: Forced Expiratory Volume1
CCSP: Club Cell Secretory Protein
BMI: Body Mass Index
RDS: Respiratory Distress Syndrome
PICU: Pediatric Intensive Care Unit
GSHPx: Glutathione peroxidase
Th: T-helper
FFQ: Food Frequency Questionnaire
EPA: Eicosapentaenoic Acid
DHA: Docosahexaenoic Acid
CRP: C-Reactive-Protein
AST: Aspartate Transaminase
LDH: Lactate Dehydrogenase
CK-MB: Creatine Kinase- Myoglobin Binding
